# Geriatric nutritional risk index as a prognostic marker of first-line immune checkpoint inhibitor combination therapy in patients with renal cell carcinoma: a retrospective multi-center study

**DOI:** 10.1007/s12672-023-00816-x

**Published:** 2023-11-16

**Authors:** Shogo Watari, Satoshi Katayama, Hiromasa Shiraishi, Moto Tokunaga, Risa Kubota, Norihiro Kusumi, Takaharu Ichikawa, Tomoyasu Tsushima, Yasuyuki Kobayashi, Kensuke Bekku, Motoo Araki

**Affiliations:** 1 Department of Urology, National Hospital Organization Okayama Medical Center, 1711-1 Tamasu, Kita-Ku, Okayama 701-1192 Japan; 2 Department of Urology, Okayama University Graduate School of Medicine, Dentistry and Pharmaceutical Sciences, 2-5-1 Shikata-Cho, Kita-Ku, Okayama 700-8558 Japan

**Keywords:** Geriatric Nutritional Risk Index, Immune checkpoint inhibitor, Renal cell carcinoma, Prognosis

## Abstract

**Purpose:**

This study aimed to investigate the effectiveness of the Geriatric Nutritional Risk Index (GNRI) in predicting the efficacy of first-line immune checkpoint inhibitor (ICI) combination therapy for metastatic or unresectable renal cell carcinoma (RCC) and associated patient prognosis.

**Methods:**

A retrospective study was conducted using data from 19 institutions. The GNRI was calculated using body mass index and serum albumin level, and patients were classified into two groups using the GNRI values, with 98 set as the cutoff point.

**Results:**

In all, 119 patients with clear cell RCC who received first-line drug therapy with ICIs were analyzed. Patients with GNRI ≥ 98 had significantly better overall survival (OS) (p = 0.008) and cancer-specific survival (CSS) (p = 0.001) rates than those with GNRI < 98; however, progression-free survival (PFS) did not differ significantly. Inverse probability of treatment weighting analysis showed that low GNRI scores were significantly associated with poor OS (p = 0.004) and CSS (p = 0.015). Multivariate analysis showed that the Karnofsky performance status (KPS) score was a better predictor of prognosis (OS; HR 5.17, p < 0.001, CSS; HR 4.82, p = 0.003) than GNRI (OS; HR 0.36, p = 0.066, CSS; HR 0.35, p = 0.072). In a subgroup analysis of patients with a good KPS and GNRI ≥ 98 vs < 98, the 2-year OS rates were 91.4% vs 66.9% (p = 0.068), 2-year CSS rates were 91.4% vs 70.1% (p = 0.073), and PFS rates were 39.7% vs 21.4 (p = 0.27), respectively.

**Conclusion:**

The prognostic efficiency of GNRI was inferior to that of the KPS score at the initiation of the first-line ICI combination therapy for clear cell RCC.

**Supplementary Information:**

The online version contains supplementary material available at 10.1007/s12672-023-00816-x.

## Introduction

More than 400,000 new cases of renal cell carcinoma (RCC) and more than 170,000 associated deaths occur each year worldwide (Global Cancer Observatory. International Agency for Research on Cancer, 2023). Immune checkpoint inhibitors (ICIs) are key agents used in the treatment of RCC as well as other malignancies in the modern era. ICI combination therapy involving ICI with tyrosine kinase inhibitor (TKI) or dual ICI agents is a first-line drug treatment for metastatic or unresectable RCC. The International Metastatic Renal Cell Carcinoma Database Consortium (IMDC) risk model is used to determine the appropriate treatment [1], although it is not a satisfactory predictive marker for response to treatment or prognosis. It is necessary to identify factors that can help predict the efficacy and prognosis of ICI treatment.

The Geriatric Nutritional Risk Index (GNRI) is a convenient nutritional assessment index based on the body mass index (BMI) and serum albumin level. Previous studies have reported the usefulness of GNRI in predicting the prognosis and adverse events of cancer treatment [2–7]. The factors included in the IMDC risk model were biomarkers that reflect inflammation, such as increased neutrophil and platelet counts [8]. Chronic inflammation can lead to a decline in nutritional status [9]. Therefore, it may be worthwhile to investigate whether GNRI, an indicator of nutritional status, is useful for selecting the treatment or predicting the prognoses of patients with metastatic or unresectable RCC.

Recently, the usefulness of GNRI in predicting the outcomes of drug therapy using ICIs has been reported for several cancer types [10–13]. A retrospective study analyzing 56 patients with RCC who received second-line ICI monotherapy reported that the GNRI was useful for predicting their prognosis [12]. However, the usefulness of the GNRI in the modern era remains unclear, considering that ICI combination therapy has become the accepted standard treatment for RCC. In this study, we investigated whether the GNRI of patients at the time of ICI combination therapy initiation is useful for predicting the efficacy of treatment and patient prognosis.

## Methods

### Patients

In this retrospective study, we evaluated the data collected from 19 institutions from June 25, 2022 until October 4, 2022. The patients who received first-line therapy with ICI were included in this study. Patients who did not have clear cell RCC and those for whom GNRI could not be calculated were excluded. The GNRI was calculated as 14.89 * serum albumin level + 41.7 * BMI/22. If the body weight was greater than the ideal body weight, to avoid misinterpreting obesity or edema as good nutritional status, the GNRI was calculated as 14.89 * serum albumin level + 41.7 * 1 [14]. The patients were classified into two groups using a cutoff GNRI of 98. Originally, the GNRI had four classifications: 98 or higher denotes good nutritional status; 92–98, mild nutritional risk; 82–92, moderate nutritional risk; and < 82, poor nutritional risk [15]; however, 98 was used as the cutoff in this study. This study was approved by the ethics board of Okayama University Graduate School of Medicine (research ID; 2207-02). Informed consent was obtained from all patients in an opt-out format. This study was conducted in accordance with the Declaration of Helsinki.

### Outcomes

The primary outcome was the association between nutritional status and overall survival (OS). The secondary outcomes were cancer-specific survival (CSS), progression-free survival (PFS), best response rate, and incidence of adverse effects. Every outcome was described at the physician's discretion at each institution. The starting point for each survival period was the initiation date of first-line therapy. Therapeutic response was described according to Response Evaluation Criteria In Solid Tumors, ver 1.1, and adverse events were described according to Common Terminology Criteria for Adverse Events, ver 5.0.

### Statistical analysis

The EZR software ver. 4.0.2 (Saitama Medical Center, Jichi Medical University, Saitama, Japan) was used for statistical analysis. Survival curves were represented using Kaplan–Meier curves. Hazard ratios (HRs) and 95% confidence intervals (CIs) were calculated using Cox proportional hazards analysis, and p-values were calculated using the log-rank test. Cox regression analysis was used to identify factors associated with survival and progression. The following covariates were included in the multivariate analysis: age, sex, Karnofsky performance status (KPS) score < 80, and anemia. Age was converted into a nominal variable, and the median age was set as the cutoff point for age. Anemia was defined as hemoglobin (Hb) < 13 mg/dL in males and Hb < 11 mg/dL in females. The low GNRI group had expected that a higher proportion of IMDC poor risk patients, likely affecting poor prognosis. To reduce bias due to differences in the patient background of IMDC risk, inverse probability of treatment weighting (IPTW) analysis based on the propensity scores was performed on the pre-matched cohort to assess OS, CSS and PFS using Cox regression analysis. The propensity scores were adjusted by age, sex, and IMDC risk classification. All statistical tests were two-tailed, and p-values < 0.05 indicated statistical significance.

## Results

**Fig. 1:**
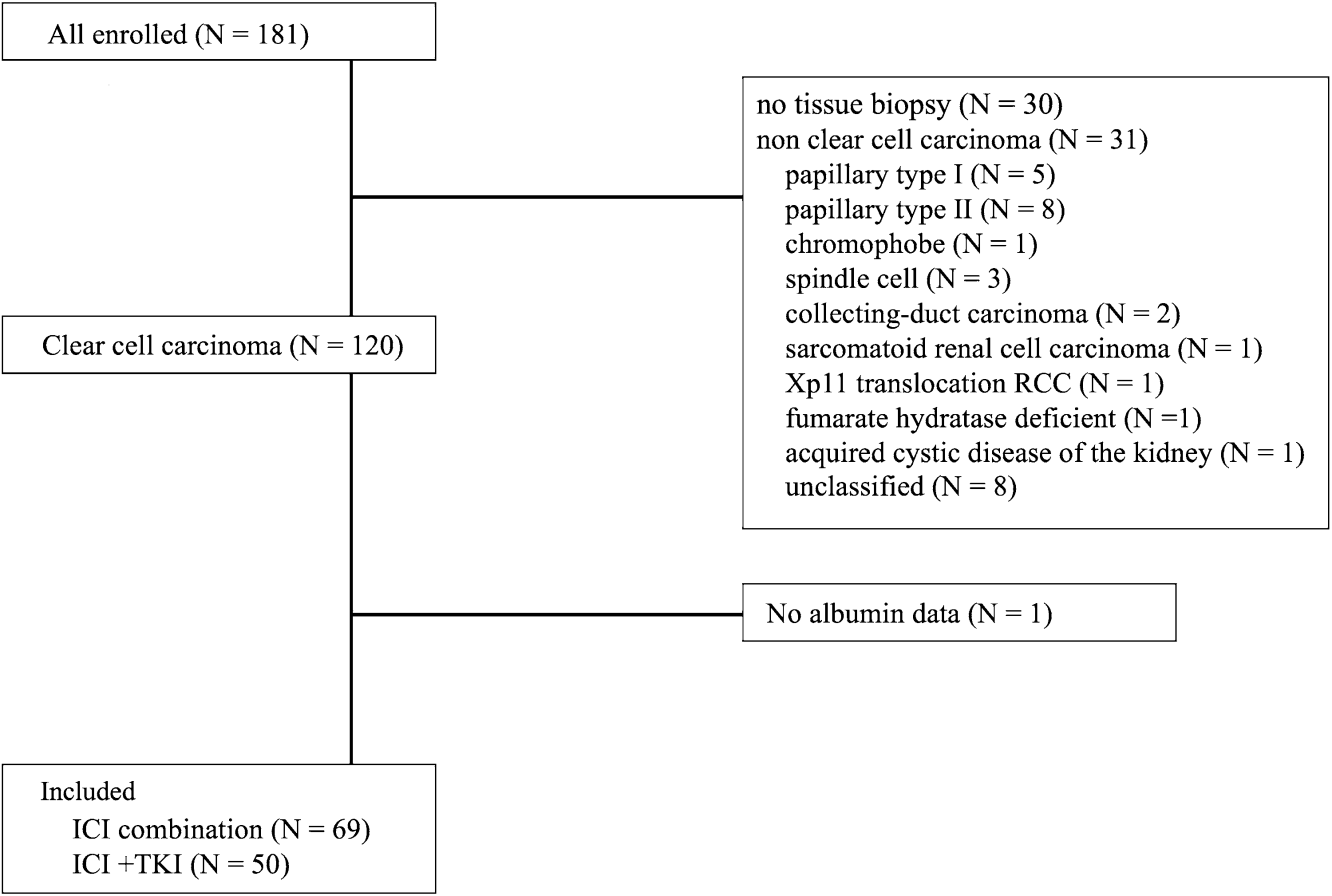
Flowchart of the patients enrolled in this study

We enrolled 181 patients who received first-line drug therapy with ICIs between August 17, 2015 and June 13, 2022. Among them, 120 had clear cell carcinoma: one patient for whom the GNRI could not be calculated owing to missing serum albumin data was excluded. Finally, 119 patients were included in the analysis (Fig. 1). Of the 119 patients, 69 received ipilimumab with nivolumab and 50 received ICI with TKI therapy. Among the latter, 40 received pembrolizumab and axitinib, five received avelumab and axitinib, and five received nivolumab and cabozantinib.

The characteristics of the patients in this study are summarized in Table 1. The group with GNRI < 98 had low BMI and serum albumin levels, and a high proportion of patients in this group had a KPS score of < 80 and IMDC poor risk category. This group also included a higher proportion of patients who did not undergo nephrectomy and had good renal function than the group with GNRI > 98. Of all the patients, 21 died, including 17 who died from RCC.

**Table 1:** Baseline characteristics of the enrolled patients

	GNRI ≧ 98	GNRI < 98
	N = 60	N = 59
Age (years)	70 (64–74)	70 (65–74)
Sex, female	15 (25)	11 (18.6)
BMI (kg/m^2^)	22.9 (21.4–25.0)	21.8 (19.4–24.8)
Serum albumin (mg/dL)	4.2 (4.0–4.4)	3.5 (3.1–3.7)
eGFR (mL/min/1.73 m^2^)	46.0 (39.8–57.0)	54.7 (38.1–71.4)
CRP (mg/dL)	0.14 (0.07–0.31)	1.41 (0.34–7.18)
GNRI	102.8 (100.3–105.8)	92.47 (84.6–95.4)
KPS < 80	2 (3)	14 (24)
cT^a^		
1–2	31 (52)	26 (44)
3	24 (40)	26 (44)
4	4 (6.7)	7 (12)
Unknown	1 (1.7)	0 (0)
Regional N positive	8 (14)	15 (25)
Metastasis		
Lung	39 (65)	42 (71)
Bone	13 (22)	16 (27)
Liver	3 (5)	6 (10)
Brain	4 (6.7)	4 (7)
Lymph node	13 (22)	12 (20)
Others	20 (33)	24 (41)
Fuhrman grade		
1–2	31 (52)	25 (42)
3	14 (24)	16 (27)
4	2 (3)	3 (5.1)
Unknown	13 (22)	15 (25)
Pathological variant	4 (6.7)	7 (12)
IMDC		
Favorable	16 (27)	3 (5)
Intermediate	39 (65)	36 (61)
Poor	5 (8.3)	20 (34)
Primary lesion resection		
Before drug treatment	46 (77)	24 (41)
After drug treatment	1 (1.7)	8 (14)
Not resected	13 (22)	27 (46)
First line		
Ipilimumab + nivolumab	29 (48)	40 (68)
Pembrolizumab + axitinib	26 (43)	14 (24)
Avelumab + axitinib	2 (3)	3 (5.1)
Nivolumab + cabozantinib	3 (5)	2 (3)
Discontinuation		
Total	28 (47)	40 (68)
PD	12 (20)	15 (25)
AE	12 (20)	19 (32)
Other	3 (5)	5 (8)
Unknown	1 (1.7)	1 (1.7)
Second line, third line	16 (27)	22 (37)
Follow-up time (months)	14 (5–25)	11 (4–21)
Death		
Renal cancer	6 (10)	15 (25)
Others	1 (3)	3 (8)

The continuous variables are expressed as medians and interquartile ranges. Nominal variables are expressed as number and percentage

*AE* adverse event, *BMI* body mass index, *CRP* C-reactive protein, *eGFR* estimated glomerular filtration rate, *GNRI* geriatric Nutritional Risk Index, *IMDC* International Metastatic Renal Cell Carcinoma Database Consortium, *KPS* Karnofsky Performance Status, *PD* progressive disease, *cT* clinical T stage

^a^The T stage was considered pathological if the patient had undergone nephrectomy; otherwise, it was considered clinical

**Fig. 2:**
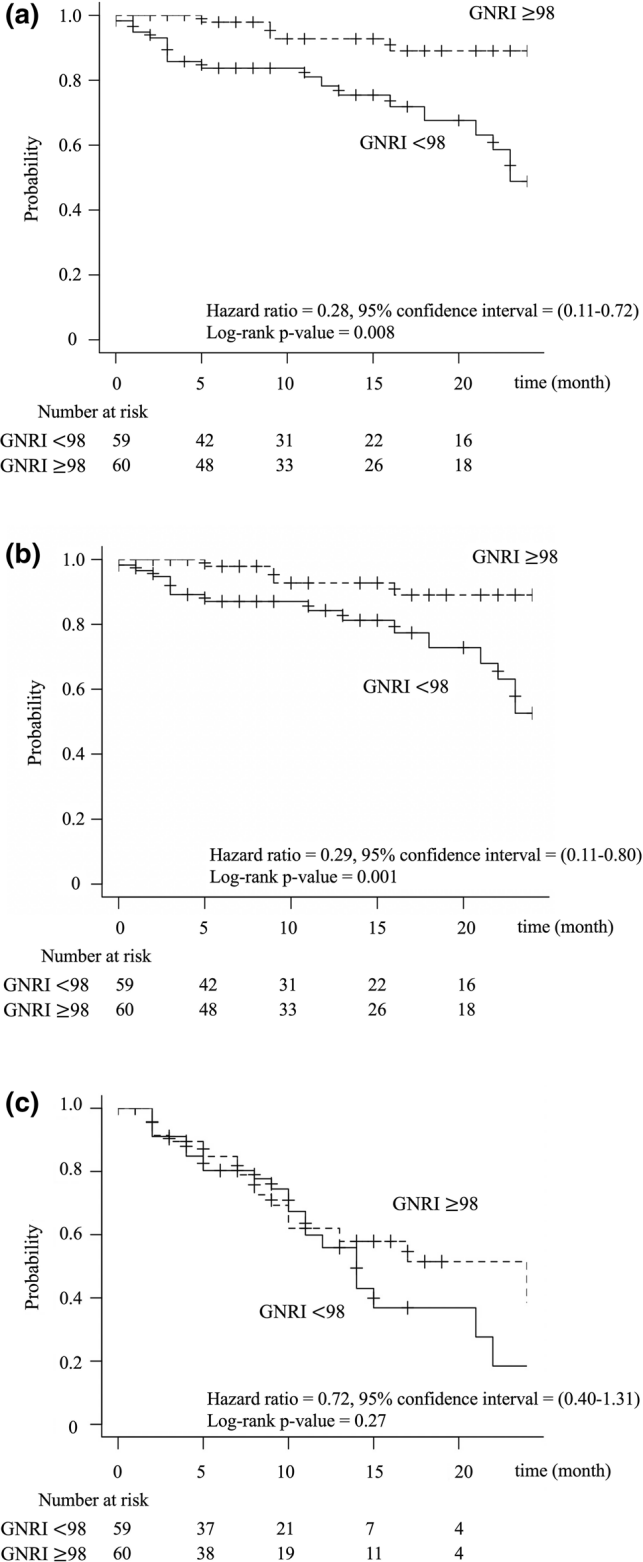
Kaplan–Meier curves according to Geriatric Nutritional Risk Index (GNRI) in all patients. **a** Overall survival, **b** cancer-specific survival, and **c** progression-free survival

Patients with GNRI ≥ 98 and < 98 had a 2-year OS of 89.1% and 48.8% (Fig. 2a), 2-year CSS of 89.1% and 52.6% (Fig. 2b) and 2-year PFS of 38.6% and 18.4% (Fig. 2c), respectively. The OS and CSS rates were significantly different between the two groups (p = 0.008 and p = 0.001), but the PFS rates were not (p = 0.27). Table 2 summarizes the IPTW analysis results and shows that low GNRI scores were significantly associated with poor OS and CSS (p = 0.004 and p = 0.015).

**Table 2:** Inverse probability of treatment weighting analysis based on the propensity scores

Parameter	OS			CSS			PFS		
	HR	95CI	p	HR	95CI	p	HR	95CI	p
GNRI ≧ 98	3.74	1.51–9.24	0.004	3.58	1.28–9.99	0.015	1.40	0.73–2.71	0.31

OR and 95% CI were obtained using multivariable regression

The propensity scores were adjusted by age, sex, and IMDC risk

*CSS* cancer-specific survival, *CI* confidence interval, *GNRI* Geriatric Nutritional Risk Index, *HR* hazard ratio, *IMDC* International Metastatic Renal Cell Carcinoma Database Consortium, *OS* overall survival, *PFS* progression-free survival, *OR* odds ratio

The results of the multivariate analysis of OS, CSS, and PFS are shown in Table 3. KPS < 80 was significantly associated with poor OS (HR, 5.99, p < 0.001) and CSS (HR, 6.19, p = 0.002). Low GNRI scores were not associated with poor OS or CSS. None of the covariates were associated with PFS. In the subgroup analysis of patients with KPS ≥ 80, patients with GNRI ≥ 98 and < 98 had 2-year OS rates of 91.4% vs 66.9% (Supplementary Figure 1a), 2-year CSS rates of 91.4% vs 70.1% (Supplementary Figure 1b), and PFS rates of 39.7% vs 21.4% (Supplementary Figure 1c), respectively.

**Table 3:** Multivariate analyses of baseline parameters for OS, CSS, PFS of all patients

Parameter									
Parameter	OS			CSS			PFS		
	HR	95% CI	p	HR	95% CI	p	HR	95% CI	p
Age > 70^a^	0.88	0.38–2.07	0.780	1.03	0.41–2.61	0.945	1.07	0.58–1.97	0.837
Sex, female	1.73	0.54–5.54	0.353	2.37	0.67–8.36	0.180	1.76	0.76–4.05	0.184
GNRI ≧ 98	0.36	0.12–1.07	0.066	0.35	0.11–1.10	0.072	0.75	0.38–1.48	0.401
KPS < 80	5.17	2.00–13.4	< 0.001	4.82	1.73–13.4	0.003	1.18	0.46–3.00	0.726
Anemia	0.52	0.15–1.78	0.296	0.48	0.12–1.91	0.297	1.06	0.48–2.37	0.884

OR and 95% CI were obtained using Multivariable regression

Anemia was defined as Hb < 11 for women and < 13 for men

*CSS* cancer-specific survival, *CI* confidence interval, *GNRI* Geriatric Nutritional Risk Index, *HR* hazard ratio, *KPS* Karnofsky Performance Status, *OS* overall survival, *PFS* progression-free survival, *OR* odds ratio

^a^The cutoffs were set at the median value

Table 4 shows the best response rates to therapy. The response rate of the GNRI ≥ 98 group was 52% and that of the GNRI < 98 group was 41%; the response rates to dual ICIs were 45% and 38% and to ICI with TKI, 58% and 47%, respectively (Supplementary Table 1). Grade 3 or higher adverse events due to dual ICI treatment occurred in 11 (37.9%) of 29 patients in the GNRI ≥ 98 group and in 16 (40%) of 40 patients in the GNRI < 98 group. Grade 3 or higher adverse events due to ICI with TKI therapy occurred in 8 of 31 patients (25.8%) in the GNRI ≥ 98 group and in 6 of 19 patients (31.6%) in the GNRI < 98 group. One Grade 5 adverse event was observed in a patient with GNRI < 98. A summary of adverse effects is provided in Supplemental Table 2.

**Table 4:** Tumor response of patients classified by their nutritional status

Tumor response	GNRI ≧ 98, n (%)	GNRI < 98, n (%)
	(N = 60)	(N = 59)
Best overall response		
CR	6 (10)	2 (3)
PR	25 (42)	22 (37)
SD	18 (30)	22 (37)
PD	8 (13)	8 (14)
Non-evaluable or missing	3 (5)	5 (8)
Objective response	31 (52)	24 (41)

*CR* complete response, *GNRI* Geriatric Nutritional Risk Index, *PD* progressive disease, *PR* partial response, *SD* stable disease

## Discussion

In this study, the GNRI was found to be a predictor of the prognosis of patients with metastatic or unresectable clear cell RCC who were treated with first-line ICI combination therapy. However, the GNRI was not identified as an independent prognostic factor, but multivariate analysis showed that KPS < 80 was associated with prognosis. The rates of response to therapy with dual ICIs and ICI with TKI were higher among patients with good nutritional status than among patients with poor nutritional status. The incidence of adverse events did not differ according to nutritional status.

The present study showed that the GNRI was able to predict the prognosis of patients with RCC who received ICI combination therapy with statistical significance. This result was consistent with those of previous studies on the usefulness of GNRI in predicting the OS of patients with metastatic renal cancer on first-line TKI treatment or second-line nivolumab monotherapy [12, 16]. Several studies have reported that albumin, a component of the GNRI, is associated with cancer immunity, as it suppresses excessive neutrophil inflammatory responses or extracellular traps involved in tumor metastasis [17–21]. Low BMI is also associated with poor prognosis of patients with malignant diseases [22]. These results support the idea that poor nutritional status, based on the combination of low albumin level and low BMI, is an effective indicator of cancer survival. In contrast, high BMI and obesity are associated with malignant tumor mortality [23]. In this study, the GNRI formula was 14.89 * serum albumin level + 41.7 * 1 if the BMI was higher than 22. Therefore, this formula is robust for obesity and allows for the evaluation of poor nutrition.

In this study, the KPS score alone and not the GNRI was associated with the OS and CSS of patients on ICI therapy, in line with the results of a previous report discussing the usefulness of the GNRI in predicting the CSS of urothelial carcinoma patients on second-line ICI monotherapy [10]. In contrast, a study of patients on second-line ICI monotherapy for lung cancer or renal cancer reported that the GNRI is a stronger predictor of prognosis than the KPS score [11, 12]. Comparisons of these data are difficult given that drug indications and patient characteristics vary across different types of cancers and treatment lines. Patients who received previous treatment might have poor nutritional status due to cachexia caused by disease progression or low albumin values which have been occasionally observed as adverse events of TKIs. It appears that the usefulness of GNRI and KPS is interchangeable depending on the type of cancer and line of treatment. The combined efficacy of KPS and GNRI in predicting prognosis has been reported among patients on second-line nivolumab monotherapy for renal cancer. However, in this study, subgroup analysis performed only for patients with favorable KPS scores showed no statistically significant results.

Furthermore, in our study, low GNRIs were not associated with the PFS, in contrast with the results of a previous study on renal or lung cancer patients on second-line ICI monotherapy [11, 12]. Some previous reports present evidence supporting the notion that albumin and BMI are associated with PFS, e.g., albumin enhances PD-L1 antitumor immunity via regulatory T cells by reducing oxidative stress and that patients with BMI > 30 on ICI treatment have high PFS rates [18, 21, 24–26]. It is possible that the response rate for first-line ICI combination treatment is higher than that for second-line nivolumab monotherapy, which is one of the reasons for the insignificant difference in PFS during the follow-up period.

The rates of response to treatment were better in the group with GNRI ≥ 98 than in the GNRI < 98 group for both dual ICI therapy and ICI with TKI therapy. The incidence of high-grade adverse events was similar between the two groups. However, we could not determine the indicators of the preferred treatment choice: dual ICI therapy or ICI with TKI. Some patients respond well to treatment with ICI and have a durable response over a long duration [27, 28]. The predictors of durable responses are still unknown. The IMDC risk model was used to select the treatment, but it did not predict durable response. The identification of useful biomarkers for treatment selection remains challenging.

This study had a few limitations. The data were collected from multiple institutions; therefore, we were not able to obtain homogenous data. The details of adverse events were not available. Unfortunately, there was one death due to immune-related myocarditis, and its association with the GNRI was unclear. We used a cutoff GNRI of 98, while previous studies used a cutoff of 92 [11, 12]. However, the cutoff point of 92 did not reveal any new significant results. In the present study, we found that poor nutritional status led to poor prognosis after ICI combination therapy for metastatic or unresectable RCC. Although this is a retrospective study, the findings provide valuable new insights into the prognoses of patients with RCC who are treated with ICIs.

## Conclusion

The prognostic efficiency of GNRI was inferior to that of the KPS score for patients who had initiated first-line therapy for clear cell RCC and were treated with dual ICIs and ICI with TKI. The usefulness of the GNRI in predicting the prognosis and antitumor efficacy of treatment using ICIs may vary according to the type of cancer or line of treatment.

### Supplementary Information

Additional file 1

Additional file 2

## Data Availability

The data related to this study are available from the corresponding author upon reasonable request.
