# Supplementary material for: Geriatric nutritional risk index as a prognostic marker of first-line immune checkpoint inhibitor combination therapy in patients with renal cell carcinoma: a retrospective multi-center study
Source: Discov Oncol. 2023 Nov 16;14:204. doi: 10.1007/s12672-023-00816-x (PMC10651608; doi:10.1007/s12672-023-00816-x)
Supplement: Supplementary file 2 — Additional file 2 [file 12672_2023_816_MOESM2_ESM.docx]

Table S1. Tumor response of patients listed by therapeutic agent

| Tumor response | GNRI ≧98, n (%) | | | | GNRI <98, n (%) | | | |  |  |  |  |
| --- | --- | --- | --- | --- | --- | --- | --- | --- | --- | --- | --- | --- |
|  | ICI combination | | ICI+TKI | | ICI combination | | ICI+TKI | |  |  |  |  |
|  | (n = 29) | | (n = 31) | | (n = 40) | | (n = 19) | |  |  |  |  |
| Best overall response |  |  |  |  |  |  |  |  |  |  |  |  |
| CR | 2 | (6.9) | 4 | (13) | 1 | (2.5) | 1 | (5.3) |  |  |  |  |
| PR | 11 | (38) | 14 | (45) | 14 | (35) | 8 | (42) |  |  |  |  |
| SD | 7 | (24) | 11 | (35) | 15 | (38) | 7 | (37) |  |  |  |  |
| PD | 7 | (24) | 1 | (3.2) | 6 | (15) | 2 | (11) |  |  |  |  |
| Non-evaluable or missing | 2 | (6.9) | 1 | (3.2) | 4 | (10) | 1 | (5.3) |  |  |  |  |
| Objective response | 13 | (45) | 18 | (58) | 15 | (38) | 9 | (47) |  |  |  |  |

CR, complete response; GNRI, Geriatric Nutritional Risk Index; ICI, immune checkpoint inhibitor; PD, progressive disease; PR, partial response; SD, stable disease; TKI, tyrosine kinase inhibitor

Table S2. Adverse events of patients classified by nutritional status

Table S2a. Immune checkpoint inhibitor combination

| AEs | GNRI ≧98 (n = 29), n (%) | | | | | GNRI <98 (n = 40), n (%) | | | | | |  | |  | |  | |  | |  | |  | |  | |  | |  | |  | |  | |  | |
| --- | --- | --- | --- | --- | --- | --- | --- | --- | --- | --- | --- | --- | --- | --- | --- | --- | --- | --- | --- | --- | --- | --- | --- | --- | --- | --- | --- | --- | --- | --- | --- | --- | --- | --- | --- |
|  | all | | | G3-4 | | all | | | G3-4 | | |  | | | |  | | | |  | | | |  | |  | |  | |  | |  | |  | |
| Rash | 10 | (35) | 0 | | (0) | 9 | (23) | 0 | | (0) |  | |  | |  | |  | |  | |  | |  | |  | |  | |  | |  | |  | |  |
| Hypothyroidism | 6 | (21) | 0 | | (0) | 0 | (0) | 0 | | (0) |  | |  | |  | |  | |  | |  | |  | |  | |  | |  | |  | |  | |  |
| Hepatitis | 5 | (17) | 4 | | (14) | 6 | (15) | 4 | | (10) |  | |  | |  | |  | |  | |  | |  | |  | |  | |  | |  | |  | |  |
| Pneumonitis | 2 | (6.9) | 1 | | (3.5) | 4 | (10) | 3 | | (7.5) |  | |  | |  | |  | |  | |  | |  | |  | |  | |  | |  | |  | |  |
| Adrenal insufficiency | 2 | (6.9) | 2 | | (6.9) | 3 | (7.5) | 2 | | (5) |  | |  | |  | |  | |  | |  | |  | |  | |  | |  | |  | |  | |  |
| Rhabdomyolysis | 2 | (6.9) | 1 | | (3.5) | 0 | (0) | 0 | | (0) |  | |  | |  | |  | |  | |  | |  | |  | |  | |  | |  | |  | |  |
| Hyperthyroidism | 1 | (3.5) | 0 | | (0) | 5 | (13) | 1 | | (2.5) |  | |  | |  | |  | |  | |  | |  | |  | |  | |  | |  | |  | |  |
| Diarrhea | 1 | (3.5) | 0 | | (0) | 3 | (7.5) | 0 | | (0) |  | |  | |  | |  | |  | |  | |  | |  | |  | |  | |  | |  | |  |
| Arthritis | 1 | (3.5) | 0 | | (0) | 2 | (5) | 1 | | (2.5) |  | |  | |  | |  | |  | |  | |  | |  | |  | |  | |  | |  | |  |
| Colitis | 1 | (3.5) | 1 | | (3.5) | 1 | (2.5) | 0 | | (0) |  | |  | |  | |  | |  | |  | |  | |  | |  | |  | |  | |  | |  |
| Hypopituitarism | 1 | (3.5) | 0 | | (0) | 1 | (2.5) | 1 | | (2.5) |  | |  | |  | |  | |  | |  | |  | |  | |  | |  | |  | |  | |  |
| Myocarditis | 1 | (3.5) | 0 | | (0) | 1 | (2.5) | 0 | | (0) |  | |  | |  | |  | |  | |  | |  | |  | |  | |  | |  | |  | |  |
| Fatigue | 1 | (3.5) | 1 | | (3.5) | 0 | (0) | 0 | | (0) |  | |  | |  | |  | |  | |  | |  | |  | |  | |  | |  | |  | |  |
| Hemophagocytic syndrome | 1 | (3.5) | 1 | | (3.5) | 0 | (0) | 0 | | (0) |  | |  | |  | |  | |  | |  | |  | |  | |  | |  | |  | |  | |  |
| Shingles | 1 | (3.5) | 0 | | (0) | 0 | (0) | 0 | | (0) |  | |  | |  | |  | |  | |  | |  | |  | |  | |  | |  | |  | |  |
| Myasthenia gravis | 1 | (3.5) | 0 | | (0) | 0 | (0) | 0 | | (0) |  | |  | |  | |  | |  | |  | |  | |  | |  | |  | |  | |  | |  |
| Neuropathy | 1 | (3.5) | 0 | | (0) | 0 | (0) | 0 | | (0) |  | |  | |  | |  | |  | |  | |  | |  | |  | |  | |  | |  | |  |
| Acute kidney injury | 0 | (0) | 0 | | (0) | 2 | (5) | 2 | | (5) |  | |  | |  | |  | |  | |  | |  | |  | |  | |  | |  | |  | |  |
| Pancreatitis | 0 | (0) | 0 | | (0) | 2 | (5) | 1 | | (2.5) |  | |  | |  | |  | |  | |  | |  | |  | |  | |  | |  | |  | |  |
| Salivary gland fistula | 0 | (0) | 0 | | (0) | 1 | (2.5) | 0 | | (0) |  | |  | |  | |  | |  | |  | |  | |  | |  | |  | |  | |  | |  |
| Encephalopathy | 0 | (0) | 0 | | (0) | 1 | (2.5) | 1 | | (2.5) |  | |  | |  | |  | |  | |  | |  | |  | |  | |  | |  | |  | |  |

If multiple adverse effects occur in one person, each is described. Grades of adverse events are described in accordance with the CTCAE v5.0. AEs, adverse events; GNRI, Geriatric Nutritional Risk Index; G, grade

Table S2b. Immune checkpoint inhibitor + tyrosine kinase inhibitor

| AEs | GNRI ≧98 (n = 31), n (%) | | | | | GNRI < 98 (n = 19), n (%) | | | | | |  | | |
| --- | --- | --- | --- | --- | --- | --- | --- | --- | --- | --- | --- | --- | --- | --- |
|  | all | | | G3-4 | | all | | | G3-4 | | |  | |  |
| Palmer-plantar erythrodysesthesia syn. | 7 | (23) | 0 | | (0) | 1 | (5.3) | 0 | | (0) |  | |  |  |
| Hypertension | 5 | (16) | 1 | | (3.2) | 2 | (11) | 0 | | (0) |  | |  |  |
| Hypothyroidism | 5 | (16) | 0 | | (0) | 1 | (5.3) | 1 | | (5.3) |  | |  |  |
| Fatigue | 4 | (13) | 0 | | (0) | 2 | (11) | 0 | | (0) |  | |  |  |
| Adrenal insufficiency | 3 | (9.7) | 2 | | (6.5) | 1 | (5.3) | 0 | | (0) |  | |  |  |
| Hepatitis | 3 | (9.7) | 2 | | (6.5) | 2 | (11) | 0 | | (0) |  | |  |  |
| Hyperthyroidism | 3 | (9.7) | 1 | | (3.2) | 0 | (0) | 0 | | (0) |  | |  |  |
| Dysarthria | 3 | (9.7) | 0 | | (0) | 0 | (0) | 0 | | (0) |  | |  |  |
| Colitis | 2 | (6.5) | 1 | | (3.2) | 0 | (0) | 0 | | (0) |  | |  |  |
| Myocarditis | 1 | (3.2) | 1 | | (3.2) | 1 | (5.3) | 1* | | (5.3) |  | |  |  |
| Uveitis | 1 | (3.2) | 1 | | (3.2) | 0 | (0) | 0 | | (0) |  | |  |  |
| Platelet count decrease | 1 | (3.2) | 0 | | (0) | 0 | (0) | 0 | | (0) |  | |  |  |
| Arthritis | 1 | (3.2) | 0 | | (0) | 0 | (0) | 0 | | (0) |  | |  |  |
| Acute kidney injury | 1 | (3.2) | 0 | | (0) | 2 | (11) | 1 | | (5.3) |  | |  |  |
| Hoarseness | 1 | (3.2) | 0 | | (0) | 0 | (0) | 0 | | (0) |  | |  |  |
| Pneumonitis | 1 | (3.2) | 0 | | (0) | 1 | (5.3) | 1 | | (5.3) |  | |  |  |
| Rash | 1 | (3.2) | 0 | | (0) | 0 | (0) | 0 | | (0) |  | |  |  |
| Hypopituitarism | 0 | (0) | 0 | | (0) | 1 | (5.3) | 1 | | (5.3) |  | |  |  |
| Hyperglycemia | 0 | (0) | 0 | | (0) | 1 | (5.3) | 1 | | (5.3) |  | |  |  |
| Mucositis oral | 0 | (0) | 0 | | (0) | 1 | (5.3) | 0 | | (0) |  | |  |  |
| Diarrhea | 0 | (0) | 0 | | (0) | 1 | (5.3) | 0 | | (0) |  | |  |  |
| Infusion reaction | 0 | (0) | 0 | | (0) | 1 | (5.3) | 0 | | (0) |  | |  |  |

If multiple adverse effects occur in one person, each is described. Grades of adverse events are described in accordance with the CTCAE v5.0. *; Grade 5 adverse event included. AEs, adverse events; GNRI, Geriatric Nutritional Risk Index; G, grade
